# Hypothalamic transcriptome analysis reveals male-specific differences in molecular pathways related to oxidative phosphorylation between Iberian pig genotypes

**DOI:** 10.1371/journal.pone.0272775

**Published:** 2022-08-16

**Authors:** Ana Heras-Molina, Yolanda Núñez, Rita Benítez, José Luis Pesántez-Pacheco, Consolación García-Contreras, Marta Vázquez-Gómez, Susana Astiz, Beatriz Isabel, Antonio González-Bulnes, Cristina Óvilo

**Affiliations:** 1 Department of Animal Breeding, INIA-CSIC, Madrid, Spain; 2 Department of Animal Production, Veterinary Faculty, UCM, Madrid, Spain; 3 Department of Animal Reproduction, INIA-CSIC, Madrid, Spain; 4 School of Veterinary Medicine and Zootechnics, Faculty of Agricultural Sciences, UC, Cuenca, Ecuador; 5 Nutrition and Obesities: Systemic Approaches Research Unit (NutriOmics), INSERM, Sorbonne Université, Paris, France; 6 Department of Animal Production, Veterinary Faculty, UCH-CEU, Valencia, Spain; University of Bologna, ITALY

## Abstract

The hypothalamus is implicated in controlling feeding and adiposity, besides many other physiological functions, and thus can be of great importance in explaining productive differences between lean and fatty pig breeds. The present study aimed to evaluate the hypothalamic transcriptome of pure Iberian (IBxIB) and Large White x Iberian crossbreds (IBxLW) at 60 days-old, produced in a single maternal environment. Results showed the implication of gender and genotype in the hypothalamic transcriptome, with 51 differentially expressed genes (DEGs) between genotypes and 10 DEGs between genders. Fourteen genotype by sex interactions were found, due to a higher genotype effect on transcriptome found in males. In fact, just 31 DEGs were identified when using only females but 158 using only males. A higher expression of genes related to mitochondrial activity in IBxIB male animals (*ND3*, *ND4*, *ND5*, *UQCRC2* and *ATP6*) was found, which was related to a higher oxidative phosphorylation and greater reactive oxygen species and nitric oxide production. IBxLW male animals showed higher expression of *SIRT3* regulator, also related to mitochondrial function. When females were analysed, such differences were not found, since only some differences in genes related to the tricarboxylic acid cycle. Thus, the results indicate a significant effect and interaction of the breed and the sex on the hypothalamic transcriptome at this early age.

## Introduction

Currently, intensive pig production relies on lean pig breeds, such as Large White, since these animals have been highly selected for meat production and, therefore, have rapid growth, high feed efficiency and high prolificacy [1, 2]. On the other hand, fatty breeds, like the Iberian pig, are rustic animals with limited genetic selection, traditionally reared in an extensive ecosystem (*dehesa*). Thus, fatty breeds are less productive, since they are smaller in size, have a longer productive cycle, slower growth rate and higher food intake and adiposity rates [3, 4]. Nowadays, there is an increasing interest in fatty breeds to produce high-quality, dry-cured meat products. These differences between breeds allow us to obtain meat products with different qualities [5, 6] and to study the pig as a biomedical model for different diseases [7], such as obesity or type 2 diabetes [8, 9].

The hypothalamus plays a major role in animal’s growth and adiposity since its functions include the control of voluntary feed intake and metabolism by the integration of various signals from the peripheral tissues (through energy and nutrient sensing mechanisms) and the generation of optimal answers [10–12]. Therefore, it has important implications in pig productive and reproductive parameters [13], with the gene expression of this organ having been extensively studied to understand pig response to stress [14], pig phenotypic differences between sexes [15, 16] and the hypothalamic implication on growth, fattening and metabolism [10, 17, 18]. Various studies have investigated the possible differences in gene expression at the hypothalamus level between pig breeds [19, 20]. However, animals from those previous studies were gestated in their corresponding lean or fatty mother, so the maternal and prenatal implications in the hypothalamic gene expression could not be left aside.

RNA-seq technique, as a next generation sequencing development, allows to perform a quantitative screening of transcription-wide gene expression patterns in individuals that, once correctly interpreted, gives important information about the metabolic events occurring, as well as the implication of different regulators in the processes [21].

Thus, the aim of the present study was to elucidate the importance of piglet’s genotype and sex on the hypothalamic transcriptome at early growth (60 days-old), independently of confounding factors related to prenatal (maternal) and early postnatal environment. This was done by using a similar approach as previous research from our group performed at fetal stages [22, 23]. This approach consists of inseminate pure Iberian sows with heterospermic semen (from Iberian and Large White boars), so two genotypes were obtained in the same maternal environment and maintained under the same conditions during postnatal development. The phenotype analysis of the animals used in the present study can be found in [24].

## Material and methods

### Ethic statement

The experiment was assessed and approved by the INIA Committee of Ethics in Animal Research (report CEEA 2013/036) and subsequently by the regional competent authority (report PROEX114/16), according to the Spanish Policy for Animal Protection (RD 53/2013), which meets the European Union Directive 2010/63/UE on the protection of research animals.

### Animals, experimental design and sampling

The study involved a total of 143 piglets born from 16 purebred Iberian sows at the farm Ibericos de Arauzo S.L. (Zorita de la Frontera, Salamanca, Spain). Pregnancies were obtained after cycle synchronization with altrenogest (Regumate, MSD, Boxmeer, The Netherlands) and insemination with heterospermic seminal doses achieved by mixing semen from two purebred Iberian and two purebred Large White boars. In brief, immediately after collection, the ejaculates obtained from these males were evaluated for semen quality (sperm concentration, morphology and motility), mixed at equal viable spermatozoa concentrations for Iberian and Large White fractions and aliquoted into 80 mL doses containing 6x10^9^ viable spermatozoa. All sows and boars were previously genotyped by pyrosequencing to confirm homozygosity for LEPRc.1987T (for the Iberian genotype) and LEPRc.1987C (for the Large White genotype), since this gene mutation is fixed in the Iberian pig [25]. The sows were fed a standard grain-based diet (89.9% of dry matter, 13% of crude protein, 2.6% of fat and 2.2 Mcal/kg of metabolizable energy; S1 Table) adjusted to fulfil individual pregnancy and lactation requirements based on data from the National Research Council [26].

A total of 104 piglets were purebred Iberian (homozygous TT for LEPRc.1987 marker; IBxIB; 51 females and 53 males) whilst 39 were crossbred Iberian x Large White (heterozygous CT for LEPRc.1987 IBxLW; 20 females and 19 males). During the first week of life, male piglets were castrated, following the RD 1135/2002, under farm’s animal handling practices. All piglets remained with sows in individual pens until weaning at the age of 21 days-old, when they were moved to collective pens and fed with a standard diet (89.5% of dry matter, 15% of crude protein, 4% of fat and 2.4 Mcal/kg of metabolizable energy; S1 Table) adjusted to fulfill growing requirements. At 60 days-old, a group of 67 piglets were selected by a representative body weight and size (avoiding outlier animals in terms of weight and body size higher or lower than mean ± 1 SD) from all litters, and were euthanized by stunning and exsanguination in compliance with RD 53/2013. The hypothalamus of 20 animals, 10 IBxIB (5 females and 5 males) and 10 IBxLW (5 females and 5 males) were obtained randomly from 10 litters by the selection of 1 purebred and 1 crossbred from each litter (therefore, no full siblings were obtained). The hypothalami were immediately snap-frozen in liquid nitrogen and maintained at -80°C until further use. The piglets from which hypothalamus were selected, were chosen randomly employing 10 litters and selecting randomly one purebred and one crossbred from each litter, so no full siblings were employed, as littermates came from different fathers (Iberian or Large White boar).

### Phenotypic data analysis

Phenotypic data was recorded as described in [24]. Phenotypic data corresponding to the animals included in the transcriptome study were analyzed using SPSS 25.0^®^ (IBM Corp., Armonk, NY, USA). Verification of normal distribution was done with a Shapiro test. The equality of variance was studied with a F-test. Effects of genotype (IBxIB *vs*. IBxLW) sex (female *vs*. male) and its interaction on developmental traits, adiposity, fatty acid composition, oxidative stress and metabolic status were assessed using two-way ANOVA and t-student or Mann Whitney test.

### RNA isolation, library construction and sequencing

Total RNA was extracted from the 20 hypothalamus samples using the RiboPure RNA purification kit (Ambio, TX, USA) according to the manufacturer’s protocols. RNA was quantified using a Nano-Drop-100 spectrophotometer (Nano-Drop Technologies, Wilmington, DE, USA), and its quality was evaluated using the Agilent 2100 Bioanalyzer (Agilent Technologies, CA, USA). RNA Integrity Number (RIN) values in this study ranged from 7.70 to 9.70, with an average of 9.03. The total RNA was diluted into a concentration of 100 ng/μl, and 3 μg were submitted to the *Centro Nacional de Análisis Genómico* (CNAG-CRG; Barcelona, Spain) for stranded paired-end mRNA-seq sequencing. Libraries were prepared using the TrueSeq mRNA-Seq sample preparation kit (Illumina Inc., Cat #RS-100-0801, San Diego, CA, USA), according to the manufacturer’s protocol. Each library was paired-end sequenced (2 x 75bp) by using TruSeq SBS Kit v3-HS in a HiSeq2000 platform (Illumina, Inc). The raw data was downloaded from CNAG servers and treated accordingly.

### Bioinformatic analysis

FastQC program version 0.11.8 [27] was used to assess the quality of raw sequencing data obtained from the 20 hypothalamic samples. TrimGalore version 0.5.0 [28] was used to qualitative trim data with default settings and to remove the sequencing adaptors and poly A and poly T tails (stringency of 6 bp, -s 6), keeping only paired-end reads where both pairs were longer than 40 bps and that had an optimal Phred Score (Q > 20). Filtered reads were mapped against the pig reference genome Sscrofa11.1 /Ensembl release 94 using HISAT2 version 2.1.0 [29]. Samstools-1.9 [30] was used to convert the SAM files obtained in the previous step into BAM archives. Read counting and merging was performed with HTSeq-count version 0.11.1 [31].

### Differential expression analysis

The files with the counts of reads’ number mapped to each gene obtained were analyzed with DESeq2 R package [32] using R 4.0.3. A filtering of the Differential Expressed Genes (DEGs) was obtained under the following two criteria: a Fold Change value (FC) ≥ 1.2 and a Benjami-Holchberg adjusted *p* value < 0.1. DESeq2 software supports more complex experimental designs in addition to two-groups setups. Using this software, RNA-seq read counts were modelled by generalized linear models, including the genotype and sex effects, the genotype effect within each sex and a full model including genotype, sex and the genotype by sex interaction effects. The comparison of the different DEGs obtained in each analysis was performed using a Venn diagram calculated and draw in http://bioinformatics.psb.ugent.be/webtools/Venn/.

Due to anormal results in one of the individuals (S1 Fig), the DESeq2 analyses were finally performed with the hypothalamus of 19 out of the 20 samples taken (9 from IBxIB piglets; 5 females and 4 males, and 10 from IBxLW piglets, 5 females and 5 males).

### Functional interpretation analysis

An enrichment analysis based on the functional annotation of the differentially expressed genes was performed using the Ingenuity Pathway Analysis software (IPA) [33] and the Search Tool for the Retrieval of Interacting Genes/Proteins (STRING) database version 11.0 [34]. In both cases, the different list of DEGs obtained with DESeq2 were uploaded into the software to determine activated pathways, functions, regulator activity or gene correlations [35, 36].

### Result validation by quantitative PCR (qPCR)

RNA obtained from the 19 animals employed for RNA-seq was used to perform the technical validation of five genes affected by genotype and/or sex (*GRPEL2*, *PDKZ1*, *SGCA*, *TBCD* and *UQCRC2*). The genes *GAPDH* and *ACTB* were selected for normalization after testing their stability with Genorm software (0.271 < M < 0.363) [37]. Additional information on the selected genes and amplification primers can be found in S2 Table. Primers were designed using Primer Select Software (DNASTAR, Madison, WI, USA) from the available ENSEMBL sequences. Quantitave PCR (qPCR) was performed as previously described [37].

The technical validation was performed by studying the Pearson correlation between the expression values obtained from RNA-Seq data and the normalized gene expression data obtained by RT-qPCR. To validate the global RNA-Seq results, the concordance correlation coefficient (CCC) [37] was calculated between the FC values estimated from RNA-Seq and qPCR expression measures for the five genes. Statistical analysis was performed with a linear model fitting genotype, sex and the genotype by sex interaction as fixed effects, and litter as random effect. All the analyses were performed using MIXED procedure of SAS 9.4 (SAS Inst. Inc., Cary, NC, USA). The model used was:

$${\text{y}}_{\text{ijkl}}={\text{Genotype}}_{\text{i}}+{\text{Sex}}_{\text{j}}+{\left(\text{Genotype}\;\text{x}\;\text{Sex}\right)}_{\text{ij}}+{\text{Litter}}_{\text{k}}+{\text{e}}_{\text{ijkl}}$$

Being y the qPCR expression result for each gene; i = 2 levels (pure Iberian pig and Iberian x Large White crossbred), j = 2 levels (female and male); k = 10 levels (number of litters).

## Results and discussion

In a previous study, we assessed the phenotypic data of the animals used in the present research [24] corresponding to the whole generated population. Firstly, from birth to weaning, body measures and weight were taken to ascertain development during lactation. After selecting 67 piglets, plasma metabolic and pro/antioxidant indexes and fatty acid composition of various tissues, as well as postnatal average daily weight gain and growth was assessed at different time-points.

In brief, IBxLW piglets were significantly heavier and larger than their IBxIB counterparts, with differences in body-weight and body-length being more prominent between males than between females. IBxLW showed higher meat content, whereas IBxIB animals had higher adipose tissue content in terms of both, subcutaneous and intramuscular fat, with higher MUFA and SFA content and less PUFA. Organs were also heavier in IBxLW piglets, but some ratios, such as brain/head ratio, were higher in IBxIB animals. At 60 days old, antioxidant capacity and lipid peroxidation levels were also different between genotypes, with the first being affected by a genotype by sex interaction (both IBxIB females and IBxLW males had greater values than their same-genotype counterparts) and the latter being higher in the IBxLW group. Fructosamine was also affected by a genotype by sex interaction, and lactate levels were significantly higher in IBxLW piglets. Those results were also found in the 19 piglets selected in the present study (Table 1 and S3 Table), demonstrating the representative nature of the sample.

**Table 1:** Most important phenotypic differences (mean ± S.E.M.) between 60 days-old pure Iberian piglets (IBxIB) and Iberian x Large White crossbreds (IBxLW) selected for the present study from the ones analysed in the previous study [2,4].

Measure	IBxIB	IBxLW	p-value
**Biparietal diameter (cm)**	7.54 ± 0.47	8.01 ± 0.39	0.026
**Trunk length (cm)**	61.2 ± 5.43	67.0 ± 3.73	0.019
**Subcutaneous fat (cm)**	7.09 ± 1.71	5.77 ± 1.36	0.079
**Intramuscular fat (%)**	7.98 ± 1.49	6.55 ± 1.25	0.035
**Body Weight (kg)**	17.3 ± 2.74	21.4 ± 2.93	0.019
**ADWG (0–60 d; g/d)**	0.21 ± 0.03	0.26 ± 0.04	0.005
**Carcass weight (kg)**	11.2 ± 2.05	14.8 ± 2.40	0.007
**Heart weight (g)**	94.7 ± 10.7	134 ± 18.7	0.000
**Lungs weight (g)**	204 ± 24.7	247 ± 23.7	0.004
**Pancreas weight (g)**	35.7 ± 9.56	42.8 ± 6.98	0.080
**Spleen weight (g)**	51.1 ± 9.10	58.5 ± 7.34	0.067
**Liver weight (g)**	439 ± 54.0	492 ± 55.6	0.057
**Brain/head weight ratio**	0.034 ± 0.01	0.03 ± 0.00	0.078
**Urea (mg/dL)**	18.5 ± 5.43	13.6 ± 2.59	0.041
**LDL-c (mg/dL)**	35.6 ± 7.54	45.7 ± 14.0	0.070
**Lactate (mg/dL)**	84.4 ± 21.5	116 ± 19.5	0.008
**FRAP (μmol/ml)**	15.6 ± 8.07	31.4 ± 21.7	0.006
**MDA (μmol/L)**	0.06 ± 0.01	0.07 ± 0.01	0.013

ADWG = average daily weight gain; LDL-c = low density lipoprotein cholesterol; FRAP = ferric reducing antioxidant power assay; MDA = malondialdehyde

### Mapping and annotation

From the hypothalamic transcriptome of 20 young piglets, an average of 47.67 million reads were obtained per sample, ranging from 34.95 to 60.50. Read length was 76 bases, and quality (Phred) Score was approximately 40. GC content ranged from 49 to 56%. HISAT2 mapping resulted in a mean alignment rate of 97%, which is in accordance with previous studies by our group in muscle [38] and in adipose tissue [39].

### Genotype effect: Differential expression and functional analysis

Firstly, the differences in the gene expression between the hypothalamic samples of IBxIB (excepting the one excluded for being an outlier; 9 piglets) and the total number of samples from IBxLW piglets (10 animals) were studied (both genders together in each genotype, so these results are independent of sex). A total of 51 differentially expressed genes (DEGs) were found. Eighteen were overexpressed in IBxIB animals (log2FC < -0.26) and 33 were overexpressed in IBxLW (log2FC > 0.26). Table 2 contains the top ten differentially expressed genes (5 overexpressed in IBxIB and 5 in IBxLW piglets), whereas the full detailed list of DEGs between both groups can be found in S4 Table.

**Table 2:** Ten most significant differentially expressed genes (B-H adjusted *p* value < 0.1) calculated with DESeq2 from hypothalamic transcriptome data of 60 days-old pure Iberian and Iberian x Large White crossbred pigs.

Gene	Complete gene name	Counts		Log2FC	B-H adjusted *p* value
		IBxIB	IBxLW		
*WFIKKN1*	WAP, Follistatin/Kazal, Immunoglobulin, Kunitz And Netrin Domain Containing 1	6.21	1.62	-1.88	0.089
*CRISP1*	Cysteine-Rich Secretory Protein 1	18.73	7.58	-1.35	0.001
*FAM160B2*	Family with sequence similarity 160, member B2	24.22	12.03	-0.98	0.002
*CNOT8*	CCR4-NOT Transcription Complex Subunit 8	14.86	7.95	-0.93	0.089
*TAGAP*	T Cell Activation RhoGTPase Activating Protein	31.45	17.35	-0.85	0.011
*KY*	Kyphoscoliosis Peptidase	1.74	10.11	2.53	0.099
*CENPF*	Centromere Protein F	1.02	5.14	2.38	0.019
*PGA5*	Pepsinogen A5	1.08	5.50	2.33	0.016
*ENSSSCG00000040909*	Novel protein coding gene	2.37	8.25	1.77	0.058
*C17orf53*	Homologous Recombincation Factor With OB-Fold	3.33	9.54	1.52	0.005

IBxIB = pure Iberian pigs; IBxLW: Iberian x Large White crossbreds; FC = fold change; B-H = Benjami Holchberg

The gene showing the highest overexpression in the IBxIB group was *WFIKKN1* (log2FC = -1.88; B-H adjusted p value = 0.09). *WFIKKN1* encodes a secreted multidomain protein with implications in inhibition of proteases [40] acting as a regulator and as a potent antagonist for different growth and differentiation [41–43]. Therefore, its upregulation in IBxIB animals could be related to the lower development found in these piglets when compared to their IBxLW littermates.

Regarding IBxLW animals, *KY* was the most over-expressed gene (log2FC = 2.53, B-H adjusted p value = 0.099). It is implicated in the function, maturation and stabilization of the neuromuscular junction and, possibly, in the normal muscle growth [44]. Therefore, the finding of this gene being upregulated in IBxLW when compared to IBxIB pigs may be related to the lower growth potential of the Iberian breed [4], although further research would be needed to clarify the exact function of this gene at the hypothalamus level.

Interestingly, leptin receptor (*LEPR*) gene was not differentially expressed between genotypes. Different expression levels between lean and Iberian pigs, with the latter having significantly lower expression levels, is a usual finding in studies comparing pure Iberian and crossbreds in tissues such as muscle [23] and liver [45] at fetal stages, whereas higher expression levels of *LEP* can be found in Iberian pigs compared to other breeds [46]. Ovilo et al [17] also found differences in the expression of this gene at the hypothalamic level which were associated with a missense polymorphism in the *LEPR* gene (LEPRc.1987T/C). Allele LEPRc. 1987T, fixed in the Iberian breed is associated with a lower hypothalamic expression of the leptin receptor. This polymorphism was used for genotype confirmation in the animals studied in the present work, with Iberians being TT and crossbreds being CT, thus differential expression of the gene was expected. A possible explanation of the result obtained would be that the age could be an important factor influencing the results. However, further research in this regard should be performed to fully understand this outcome.

When the DEGs obtained in the genotype comparison employing all piglets (S4 Table) was used for functional interpretation, results with STRING software were only related to *Ribonuclease CAF1* and to the *CCR4-NOT transcription complex subunit 7/8/Pop2* implicated in the 3’ to 5’ mRNA deadenylation [47, 48], a process related to synaptic changes and plasticity and inflammatory processes [49], which is consistent with our results. Using IPA, limited results were obtained, with no differences in canonical pathways, the upstream analysis nor regulators. There were differences only in specific functions, such as *Nervous system development and function*, in which 5 molecules were implicated (SLC6A9, ADCYAPI, ITPRI, MAPK7 and LSS), with p-values ranging from 4.36x10^-2^ to 1.86x10^-3^.

### Sex effect: Differential expression and functional analysis

When differences between sexes (by the comparison of IBxIB and IBxLW females *vs*. IBxIB and IBxLW males) were analysed, 10 genes were differentially expressed (Table 3). Only *RBBP7* gene was annotated, the remaining being novel genes. Two genes were overexpressed in females (including *RBBP7*), the rest of them being overexpressed in males. No results were obtained in the functional analysis.

**Table 3:** Ten significant differentially expressed genes (B-H adjusted *p* value < 0.1) calculated with DESeq2 from hypothalamic transcriptome data of 60 days-old pure Iberian and Iberian x Large White crossbreds pigs comparing females vs males.

Gene	Complete gene name	Counts		Log2FC	B-H adjusted *p* value
		Females	Males		
*ENSSSCG00000032435*	ncRNAs	101	0	-9.25	6.43E-37
*ENSSSCG00000012178*	novel protein coding gene	0	46.76	7.92	2.63E-29
*ENSSSCG00000032661*	novel ncRNA gene	0	41.37	7.75	4.77E-28
*ENSSSCG00000012179*	novel protein coding gene	0	13.19	6.10	2.32E-15
*ENSSSCG00000026430*	novel protein coding gene	0.20	9.05	4.96	1.29E-08
*ENSSSCG00000033734*	novel protein coding gene	0	6.26	5.02	2.90E-08
*ENSSSCG00000031969*	novel protein coding gene	0	5.40	4.82	2.65E-07
*RBBP7*	RB binding protein 7, chromatin remodeling factor	154	101	-0.61	6.99E-06
*ENSSSCG00000026864*	novel protein coding gene	0	2.76	3.83	0.001
*ENSSSCG00000027046*	novel protein coding gene	0	2.16	3.44	0.085

FC = fold change; B-H = Benjami Holchberg

*RBBP7* is a nuclear protein that belongs to a highly conserved subfamily of WD-repeat proteins that can be found in many histone acetyltransferase complexes [50]. Its principal function is to regulate cell differentiation and proliferation by binding to retinoblastoma protein [51]. At the central nervous system level, low expression levels of this gene have been related to alterations in brain including Alzheimer’s disease, neuritic plaque density and Braak Staging [52, 53]. In the present study, the higher expression found in females compared to males could be related to the general higher survival rate found in female piglet when compared to male counterparts [54–56] that could be explained by a higher or earlier neural development in this sex [57].

### Genotype by sex interaction effect: Differential expression and functional analysis

Regarding the genotype by sex interaction, there were 14 qualitative significant interactions (Table 4), indicating that, even if, as stated before, sex alone had limited effect on our results, it was important when considered with the genotype effect.

**Table 4:** Genotype by sex significant (B-H adjusted p value < 0.1) qualitative interactions in gene expression calculated with DESeq2 from hypothalamic transcriptome data of 60 days-old pure Iberian and Iberian x Large White crossbred pigs.

Gene	Complete gene name	Counts				Log2FC	B-H adjusted *p* value
		IBxIB		IBxLW			
		Females	Males	Females	Males		
***ENSSSCG00000031599***	novel LincRNA gene	22.73	48.99	58.05	23.80	-2.26	0.069
***GRPEL2***	GrpE Like 2	55.65	89.60	74.30	48.17	-1.33	0.000
***TASOR***	Transcription Activation Suppressor	45.53	64.14	66.39	40.86	-1.16	0.083
***RBM12B***	RNA Binding Motif Protein 12B	74.97	110.81	99.93	72.31	-1.00	0.095
***SETD1B***	SET domain containing 1B, histone lysine methyltransferase	39.00	66.52	59.07	50.57	-0.97	0.069
***RARB***	Retinoic acid receptor beta	81.82	109.65	87.47	66.55	-0.83	0.095
***ZNF692***	Zinc Finger Protein 692	63.41	84.01	89.38	66.83	-0.81	0.095
***CELSR1***	Cadherin EGF LAG Seven-Pass G-Type Receptor 1	1206.61	1671.90	1637.50	1216.10	-0.79	0.069
***PLPP1***	Phospholipid Phosphatase 1	288.78	380.00	333.44	269.05	-0.70	0.069
***TMEM165***	Transmembrane Protein 165	450.37	562.57	546.22	406.17	-0.68	0.083
***LIN37***	Lin-37 DREAM MuvB Core Complex Component	3418.89	2848.14	3024.74	3578.30	0.46	0.084
***ZCWPW1***	Zinc Finger CW-Type And PWWP Domain Containing 1	448.72	359.95	364.73	439.84	0.55	0.083
***DYNC1H1***	Dynein Cytoplasmic 1 Heavy Chain 1	205.55	164.65	178.42	224.33	0.61	0.095
***PSAP***	Prosaposin	143.90	106.82	111.54	149.60	0.79	0.069

IBxIB = pure Iberian pigs; IBxLW: Iberian x Large White crossbreds; FC = fold change; B-H = Benjami Holchberg

Several genes were found in which the higher count values were observed in IBxIB females and in IBxLW males compared to their same genotype counterparts (*LIN37*, *ZCWPW1*, *DYNC1H1*, *PSAP*). Also, genes were detected showing higher counts in IBxIB males and IBxLW females when compared to IBxIB females and IBxLW males (*ENSSSCG00000031599*, *GRPEL2*, *TASOR*, *RBM12B*, *SETD1B*, *RARB*, *ZNF692*, *CELSR1*, *PLPP1*, *TMEM165*).

The highest fold change was observed for *GRPEL2*. This molecule is highly influenced by oxidative stress, being a “sensor”, that is capable of augment the activity of mtHsp70 in cases of high oxidative stress levels to prevent the misfolding of mitochondrial imported proteins [58, 59]. The higher expression observed in IBxIB males and IBxLW females is in accordance with the results found in the malondialdehyde (MDA) levels when analysing the phenotype of these animals [24], since IBxIB males and IBxLW females had higher MDA levels than their same genotype and opposite sex counterparts. MDA is a lipid oxidation marker [60] thus, higher oxidation levels would increase the *GRPEL2* expression and dimerization in order to control the redox state of the individual [58].

Again, no significant results were obtained when performing the functional analysis with STRING nor IPA.

### Genotype effect within sex: Differential expression and functional analysis

Phenotype analysis of the animals showed a strong sex effect, with differences between IBxIB and IBxLW males being higher than when comparing females [24]. Furthermore, a great number of genotype by sex interactions were found when performing the differential expression analysis with DESeq2. Thus, to fully understand the processes occurring in males and in females in the hypothalamus that could explain the phenotypic differences, the 19 piglets were divided according to their sex, and the genotype effect was studied in each sex separately.

When only females were studied, 31 DEGs were found according to genotype. Thirteen DEGs were overexpressed in IBxIB, and 18 in IBxLW female piglets (Table 5 and S5 Table).

**Table 5:** Ten most significant differentially expressed genes (B-H adjusted *p* value < 0.1) calculated with DESeq2 in hypothalamic transcriptome samples of pure Iberian and Iberian x Large White crossbreds females at 60 days-old.

Gene	Complete gene name	Counts		Log2FC	B-H adjusted *p* value
		IBxIB	IBxLW		
***FAM160B2***	FHF Complex Subunit HOOK Interacting Protein 2B	24.28	11.02	-1.12	0.082
***SLA-DQB1***	Swine leukocyte antigens DQB1	88.20	45.18	-0.98	0.025
**ENSSSCG00000018073**	Novel Mt tRNA	237.76	137.89	-0.78	0.016
***PGGHG***	Protein-glucosylgalactosylhydroxylysine glucosidase	385.33	235.49	-0.71	0.001
ENSSSCG00000018096	Mt tRNA	480.75	299.21	-0.69	0.020
***USP16***	Ubiquitin Specific Peptidase 16	5.37	17.24	1.82	0.025
***GYPC***	Glycophorin C	7.27	24.71	1.77	0.021
***TARBP1***	TAR (HIV-1) RNA Binding Protein 1	7.00	19.49	1.50	0.025
***TRPC4AP***	Transient Receptor Potential Cation Channel Subfamily C Member 4 Associated Protein	7.89	18.89	1.24	0.089
***SEPTIN1***	Septin 1	17.22	39.12	1.17	0.002

IBxIB = pure Iberian pigs; IBxLW: Iberian x Large White crossbreds; FC = fold change; B-H = Benjami Holchberg

*FAM160B2* (log2FC = -1.12; B-H adjusted p value = 0.082) and *USP16* (log2FC = 1.82; B-H adjusted p value = 0.025) were the genes with the highest Fold Change in IBxIB and IBxLW females, respectively. *FAM160B2*, also known as Retinoic acid induced 16 (*RAI16*), is a gene possibly engaged in the maintenance of the intestinal barrier and in the immunoprotective inflammation due to its ability to activate the MAPS/ERK and TGFB signalling pathways [61]. It has been studied at the hepatic tissue [62] and in prostate cells [63]. However, at the central nervous system (CNS) only its analogous *FAM160B1* has been studied, with results indicating that deficits of this gene are related to severe microencephaly and intellectual disability [64]. Thus, further studies for *FAM160B2* at CNS level are necessary to ascertain possible implications in its development and, therefore, survival of the animal.

On the other hand, *USP16* is implicated in T-cell mediated immunity [65], and also in the LDL receptor stability and activity [66]. The latter function could have relevant implications in the piglets of the present study. The Iberian pig, with its natural leptin resistance, shows similar characteristics to those of obese and diabetic humans, including high levels of LDL-c in blood in the later age [8, 9]. Thus, the higher expression of this gene in IBxLW females could be related to the overall lower levels of LDL-c in this group, even if at 60 days-old no significant differences were found [24].

When functional analysis was performed with the set of DEGs detected in only females, results in STRING showed a reactome pathway related to the tricarboxilic acid (TCA) cycle and respiratory electron transport, with two genes implicated (*CHD8* and *SLA-DQB1*; overexpressed in IBxIB females). Since both genes implicated in this pathway were overexpressed in IBxIB females, this result could mean higher oxidation rates in pure Iberian animals than crossbreds and, therefore, an impairment of the modulation of energy balance at the hypothalamic level [67], with possibly higher production of reactive oxygen species (ROS) during the oxidative phosphorylation reactions, since both processes are linked [68].

The genotype effect was also evaluated within males. Accordingly to their higher differences in phenotype characteristics [24], IBxIB and IBxLW males showed a greater number of DEGs than females (158 DEGs, 120 overexpressed in IBxIB and 38 in IBxLW males). Excluding a novel gene, *CDADC1* and *TBCD* were the two genes with the highest log2FC (-1.04 and 1.32, respectively; Table 6 and S6 Table).

**Table 6:** Ten most significant differentially expressed genes (B-H adjusted p value < 0.1) calculated with DESeq2 in hypothalamic transcriptome samples of 60 days-old pure Iberian and Iberian x Large White crossbred male pigs.

Gene	Complete gene name	Counts		Log2FC	B-H adjusted *p* value
		IBxIB	IBxLW		
***ENSSSCG00000035000***	Novel protein coding gene	146.62	59.89	-1.28	0.000
***CDADC1***	Cytidine And DCMP Deaminase Domain Containing 1	52.81	25.72	-1.04	0.011
***ITGA2***	Integrin Subunit Alpha 2	112.71	58.64	-0.94	0.006
***GRPEL2***	GrpE Like 2	96.58	52.04	-0.90	0.001
***EPHB4***	EPH Receptor B4	63.49	35.32	-0.85	0.049
***TBCD***	Tubulin Folding Cofactor D	33.43	83.49	1.32	0.000
***POMC***	Proopiomelanocortin	17.86	40.49	1.16	0.028
***SGCA***	Sarcoglycan alpha	70.00	149.80	1.10	0.067
***DUT***	Deoxyuridine Triphosphatase	26.59	45.53	0.77	0.071
***WDR45B***	WD Repeat Domain 45B	25.67	42.22	0.72	0.081

IBxIB = pure Iberian pigs; IBxLW: Iberian x Large White crossbreds; FC = fold change; B-H = Benjami Holchberg

*CDADC1* (or *NYD-SP15*) is a protein coding gene, highly overexpressed in IBxIB males. It has a potential inhibitory effect on cell growth [69], and also has important implications in spermatogenesis and testicular development [70]. This result is in accordance to *CRISP1* (also related to male fertility, [71]) overexpression observed in pure animals when the total number of animals (IBxIB *vs*. IBxLW) were analysed. Thus, both findings could be related to an earlier onset of puberty in IBxIB animals. However, it should be highlighted that these piglets were castrated during the first week of life, so further research is needed to confirm this hypothesis.

*TBCD*, overexpressed in IBxLW pigs, is pivotal in the correct folding of microtubulins [72]. Therefore, it plays a major role in the adequate function and development of the body, especially the nervous system, since mutations in this gene have been related to different neuropathologies [73, 74]. This outcome agrees with results obtained at the phenotype level. Although IBxLW females also overexpressed this gene, they did it in a lesser extent than males. As explained before, differences between males in body-weight and size were greater than between females [24], an outcome in which *TBCD* gene could be implicated. This would be in accordance with previous results in Iberian pigs in which a higher growth rate was seen in early development in females when compared to males in IUGR conditions [56]. The higher growth of females in the early development compared to males has also been described in humans [75] and rats [76, 77], where a different feeding behaviour between females and males controlled by the hypothalamus was hypothesized. Even though the animals selected in the present study were not IUGRs animals, it is plausible that, being a rustic breed, Iberian females may be better prepared for survival, given their importance in the species continuity. This would explain the limited differences observed in females at the transcriptome level in comparison to males, with a lower number of DEGs or, in the case of some essential genes for development as *TBCD*, with the difference in expression being less significant.

**Fig 1:**
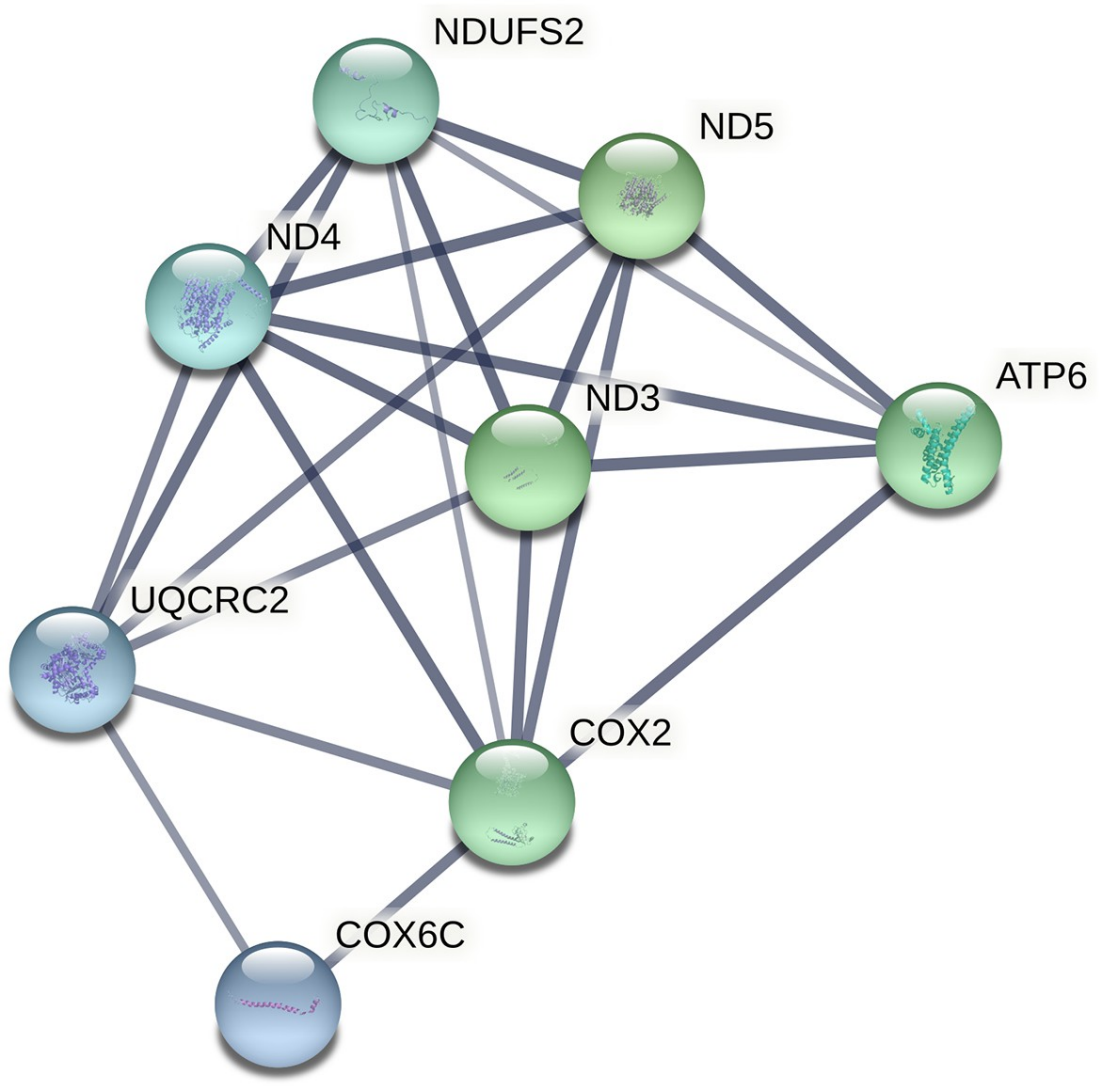
Clustering of mitochondrial DEGs related to electron transport and oxydative phosphorilation obtained with the Search Tool for the Retrieval of Interacting Genes/Proteins (STRING) software. Line thickness indicates the strenght of data suport.

The list of male-specific DEGs was analysed to study its functional implications. In this case, processes related to mitochondrial respiration, electron transport and oxidative phosphorylation showed a high relevance, with a great number of DEGs implicated in such biological functions. STRING software allowed the clustering of functionally related DEGs involved in these processes (Fig 1).

Results obtained with the software IPA also showed significant differences in functions such as Cellular Assembly and Organization, Cell-To-Cell Signalling and Interactions, Lipid Metabolism or Organismal Development (number of molecules ranging from 16 to 40, p value ranging from 6.87x10^-3^ to 3.82x10^-5^). Ten regulators showed significant z-scores (Table 7). Five of them were activated in the IBxIB genotype (negative z-score), whereas the other five were activated in IBxLW animals (positive z-score).

**Table 7:** Regulators predicted by IPA to be activated in 60 days-old pure Iberians (IBxIB; negative z-score) and in Iberian x Large White crossbred pigs (IBxLW; positive z-score). These regulators potentially control the differences in the hypothalamic transcriptome observed in males.

Upstream Regulator	Molecule Type	Predicted Activation State	Activation z-score	p-value of overlap	Target Molecules in Dataset
**ALKBH1**	Enzyme	IBxIB	-2.000	1.21E-07	*MT-ATP6*, *MT-CO2*, *MT-ND4*, *MT-ND5*
**NSUN3**	Enzyme	IBxIB	-2.000	1.21E-07	*MT-ATP6*, *MT-CO2*, *MT-ND4*, *MT-ND5*
**LDB1**	Transcription regulator	IBxIB	-2.000	1.17E-01	*CPSF7*, *EPHB4*, *GPRC5B*, *RASGRP1*
**LONP1**	Peptidase	IBxIB	-2.236	1.51E-04	*MT-ATP6*, *MT-CO2*, *MT-ND3*, *MT-ND4*, *MT-ND5*
**DAP3**	Other	IBxIB	-2.236	1.38E-08	*MT-ATP6*, *MT-CO2*, *MT-ND3*, *MT-ND4*, *MT-ND5*
**SIRT3**	Enzyme	IBxLW	2.236	1.24E-05	*MT-ATP6*, *MT-CO2*, *MT-ND3*, *MT-ND4*, *MT-ND5*
**IL10RA**	Transmembrane receptor	IBxLW	2.000	1.34E-01	*CCN1*, *NDRG2*, *POMC*, *STAT1*
**NUPR1**	Transcription regulator	IBxLW	2.449	1.15E-01	*AP5M1*, *CCN1*, *MMS22L*, *MSH6*, *SLC2A12*, *TOLLIP*
**DICER1**	Enzyme	IBxLW	2.000	1.40E-02	*CCN1*, *CCNG1*, *CDKN2B*, *ITGA2*, *MT-ND5*, *RASGRP1*
**PKM**	Kinase	IBxLW	2.213	8.38E-05	*MT-ATP6*, *MT-CO2*, *MT-ND3*, *PC*, *UQCRC2*

When analysing IBxIB vs. IBxLW males, mitochondrial gene regulators such as LONP1, ALKBH1, NSUN3 or DAP3 showed negative significant z-scores (activated in IBxIB males), and the canonical pathway Oxidative Phosphorylation showed a significant z-score (z-score = -2.121; overrepresented in IBxIB animals), implying a higher activation of this pathway in pure Iberian males. In fact, the genes implicated in the canonical pathway were the same highlighted by the STRING software. All mitochondrial genes except *NDUFS2* were overexpressed in IBxIB animals (Table 8). In fact, the hypothalamic mitochondrial gene regulation found in this research was similar to the one found in the muscle in previous studies, since these results are in accordance with recent work reporting gene expression differences between lean and fatty Serbian pig breeds analysing the muscle transcriptome of adult animals [37], in which oxidative phosphorylation was the main process affected by genotype, being activated in the muscle of the fat Mangalitsa breed.

**Table 8:** Significant (B-H adjusted *p* value < 0.1) differentially expressed genes, observed in 60 days-old pure Iberians (IBxIB) and Iberian x Large White crossbreds pigs (IBxLW) males, related to mitochondrial functions and the respiratory chain complex they belong to.

Gene	Complete gene name	Counts		log2FC	B-H adjusted *p* value	Complex
		IBxIB	IBxLW			
***ND3***	NADH dehydrogenase subunit 3	2615.91	2008.97	-0.38	0.079	I
***ND4***	NADH dehydrogenase subunit 4	16488.40	12760.69	-0.37	0.081	I
***ND5***	NADH dehydrogenase subunit	26752.54	20832.45	-0.36	0.090	I
***NDUFS2***	NADH:Ubiquinone Oxidoreductase Core Subunit S2	247.47	319.66	0.37	0.069	I
***UQCRC2***	Ubiquinol-Cytochrome C Reductase Core Protein 2	44.63	25.60	-0.81	0.061	III
***COX2***	Cytochomes C oxidase subunit 2	3505.57	2650.93	-0.40	0.081	IV
***COX6C***	Cytochomes C oxidase subunit 6	343.53	275.55	-0.31	0.095	IV
***ATP6***	Mitochondrially encoded ATP synthase membrane subunit 6	5440.89	4276.71	-0.35	0.079	V

*FC = fold change; B-H =* Benjami Holchberg

**Fig 2:**
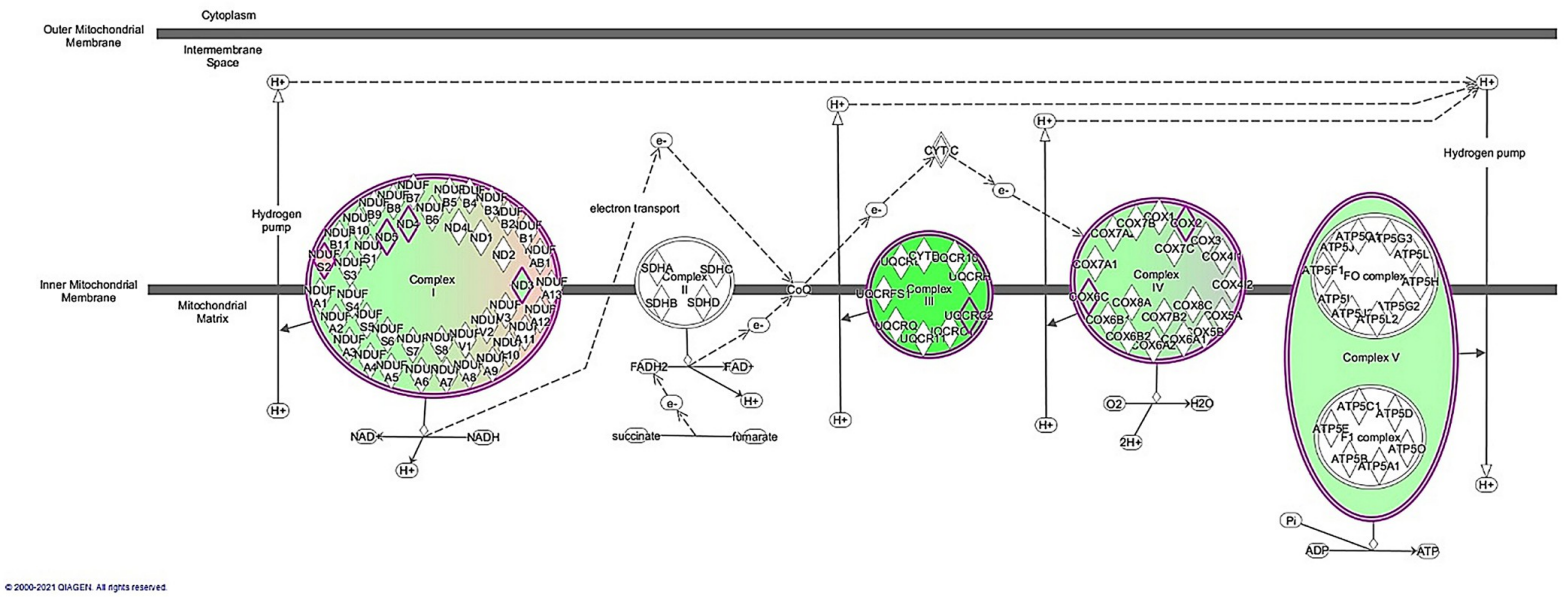
Oxidative phosphorylation pathway with genes predicted to be activated in pure Iberian (IBxIB; coloured in green) males when compared with Iberian x Large White crossbreds (IBxLW; coloured in red), obtained using Ingenuity Pathway Software (IPA).

Oxidative phosphorylation (OXPHOS) is the process in which ATP is formed because of the transfer of electrons from NADH or FADH2 to O2 by a series of electron carriers (complexes). It constitutes the major source of ATP in aerobic organisms, being performed in the inner layer of the mitochondria [78]. Fig 2 illustrates this process, with genes overexpressed in IBxIB or IBxLW males coloured in green or red, respectively. Concretely, *ND3*, *ND4* and *ND5* (overexpressed in IBxIB pigs) and *NDUFS2* (overexpressed in IBxLW pigs) are implicated in the correct function of the complex I [79], which is the major entry point for electrons. Thus, it is considered the rate-limiting step in mitochondrial respiration, playing an essential role in energy metabolism [80]. *UQCRC2* is a core protein of the complex III [81], which is overexpressed in IBxIB males. In this complex, electrons, after passing through the coenzyme Q, are transferred from one subunit to other to produce ATP [82]. Afterwards, electrons are transferred to complex IV, being the DEGs *COX2* and *COX6* (more expressed in IBxIB males) part of this complex. The electrons are transferred to oxygen and hydrogen (with high levels of energy yielded) [83], whereas protons are pumped across the membrane [84]. *ATP6* is a DEG also with higher expression levels in pure males than crossbreds involved in the complex V, the last step of the respiratory chain, which transforms ADP to ATP in the mitochondrial matrix using the energy provided in the previous steps of the process [85].

OXPHOS has been related to obesity and type 2 diabetes, being considered a key factor in the development of these disorders [86–88]. The excessive intake of nutrients (or leptin resistance states) makes the mitochondria overloaded with nutrients such as glucose and fatty acids, which increases the production of Acetil-CoA, NADH and, therefore, the proportions of electrons that enters the mitochoncrial intermembrane space [89]. This process is a major productor of reactive oxygen species (ROS; reviewed in [90]). In fact, another canonical pathway affected by genotype in males was *Production of Nitric Oxide and Reactive Oxygen Species in Macrophages* (enriched in IBxIB males when compared with same-sex piglets from the IBxLW group; z-core = -1.000). Both, nitric oxyde (NO) and ROS lead to oxydative stress [91], which is related to inflammation, adiposity, metabolic syndrome and insulin resistance [92, 93]. Specifically, the production of NO is closely associated with the hypothalamic inflammation in obese states, being mediated by macrophages [94]. These inmune cells play a major role in the diabetes progression, since they are also major producers of ROS [95]. ROS also mediates the higher differentiation of macrophages to M1 type, which are proinflammatory, and implicated in the chronic inflammation found in obese and diabetic individuals [96]. These results obtained at the hypothalamic level in the present study are in agreement with the outcomes found when comparing the muscle transcriptome of Mangalitsa and Moravka Pigs [37], in which genes related to OXPHOS and mytochondrial dysfunction were also upregulated in the fatty breed (Mangalitsa) when compared to lean ones (Moravka), even if the age, breeds and tissues are different between both studies. Thus, results seemed to be more probably related to the obesity and lean-type of each breed than the genotype itself. This fact could implicate a relation of the increased phosphorylation with obesity and diabetoid states, as seen in humans [97, 98].

**Fig 3:**
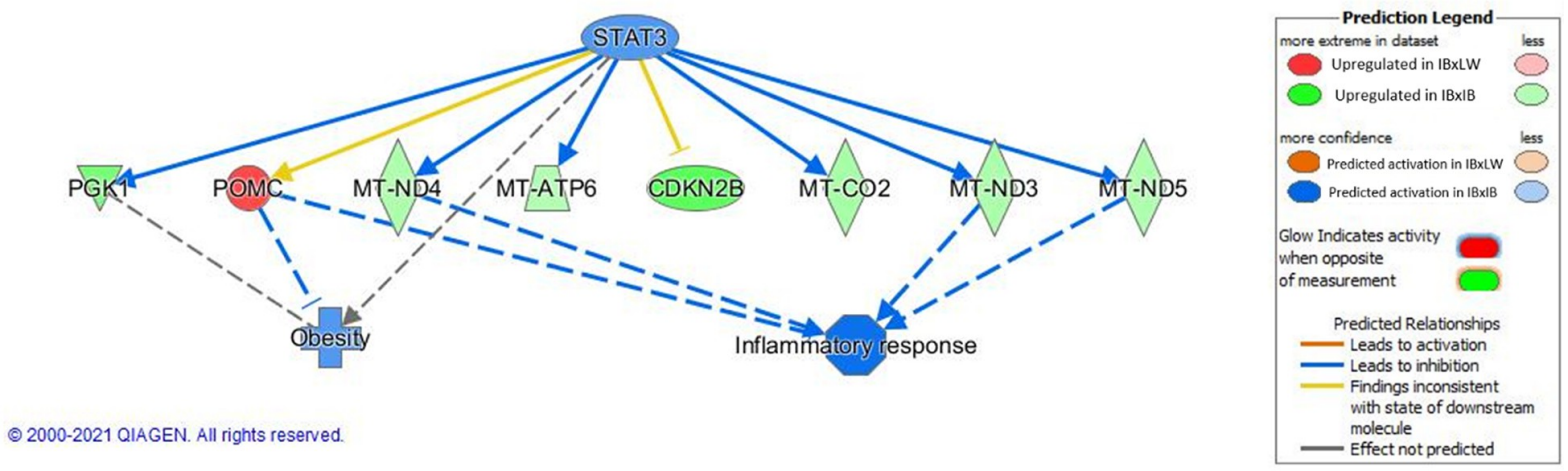
Causal network predicted for STAT3 regulator showing its relationship with obesity and inflammatory response, obtained using Ingenuity Pathway Analysis (IPA). IBxIB = pure Iberian pigs; IBxLW = Iberian x Large White crossbreds.

Results from IPA also showed potential differences in important metabolism regulators such as STAT3 (z-score = -1.000; its causal network is represented in Fig 3). The STAT is a family of transcription factors with important functions in signal regulation [99–101]. Concretely, STAT3 has multiple isoforms, and regulatory effects [102]. This regulator is being increasingly studied in the hypothalamus because of its important relation with leptin levels and, therefore, leptin resistance (as reviewed by Liu et al [103]). Thus, in response to leptin, a transcriptional cascade is activated, including STAT3, which has important effects on energy balance [104]. At the hypothalamus level, the stimulation by leptin makes STAT3 bind proopiomelanocortin (POMC) [105]. POMC is a propeptide expressed in the hypothalamus which produces in this location the rising of different substance, such as α and β-melanocyte stimulating hormones. Those hormones have anorexigenic functions, also enhancing insuling sentitivity and stimulating energy expenditure [106]. Thus, a lower POMC expression leads to severe hyperphagia and obesity in humans, mice and pig [107–109]. Obesity produces hypothalamic inflammation and higher stress, impairing, in turn, POMC function [110–112]. Accordingly to the greater ingestion capacity of the Iberian pig compared to commercial breeds [113], the *POMC* gene was overexpressed in IBxLW in comparison to IBxIB males (Table 6, red node in Fig 3). This result is in agreement with the leptin resistance syndrome, characteristic of Iberian pigs, because a reduced leptin signalling leads to a lower expression of downstream anorexigenic genes such as *POMC*, although, as explained before, no expression difference was observed at the *LEPR* gene level. Also, as represented in the figure, STAT3 has important implications in the mitochondrial gene expression and oxidative metabolism [114], which agrees with results regarding differences in oxidative stress between IBxIB and IBxLW stated before. All these outcomes are related to obesity and inflammatory response, being activated in IBxIB animals, as previously demonstrated [8, 46].

The canonical pathway *Sirtuin Signaling Pathway* was potentially enriched in IBxLW male piglets when compared with IBxIB males (z-score = 0.447). Sirtuins are a protein family highly conserved from bacteria to mammals and implicated in the regulation of many biological processes, such as stress, metabolism, development or longevity [115]. Concretely, SIRT3, a mitochondrial sirtuin [116], was detected by IPA as an upstream regulator activated in IBxLW animals (z-score = 2.236). It has been suggested that SIRT3 has important implications in mitochondrial activity regulation, especially by deacetylation of different molecules [117]. This enzyme rises during fasting or caloric restriction [118–121], and declines as response to high-fat diets or in insulin-resistant states [119, 122, 123]. Since diet in both groups was the same throughout the experiment, it is possible that the difference in SIRT3 activation is associated to the natural insulin-resistant state of the Iberian pig [8], which would explain why it is more activated in crossbreds than in pure males.

All in all, results found when only comparing males were mostly correlated with mitochondrial dysfunction (leading to higher OXPHOS and, therefore, with oxidative stress and inflammation mediated by proinflammatory macrophages), and with differences in metabolism regulated by STAT3 and sirtuins. However, further research is required to fully clarify the implications of these findings in the differences between lean and obese individuals.

### Comparative analysis of results among datasets

**Fig 4:**
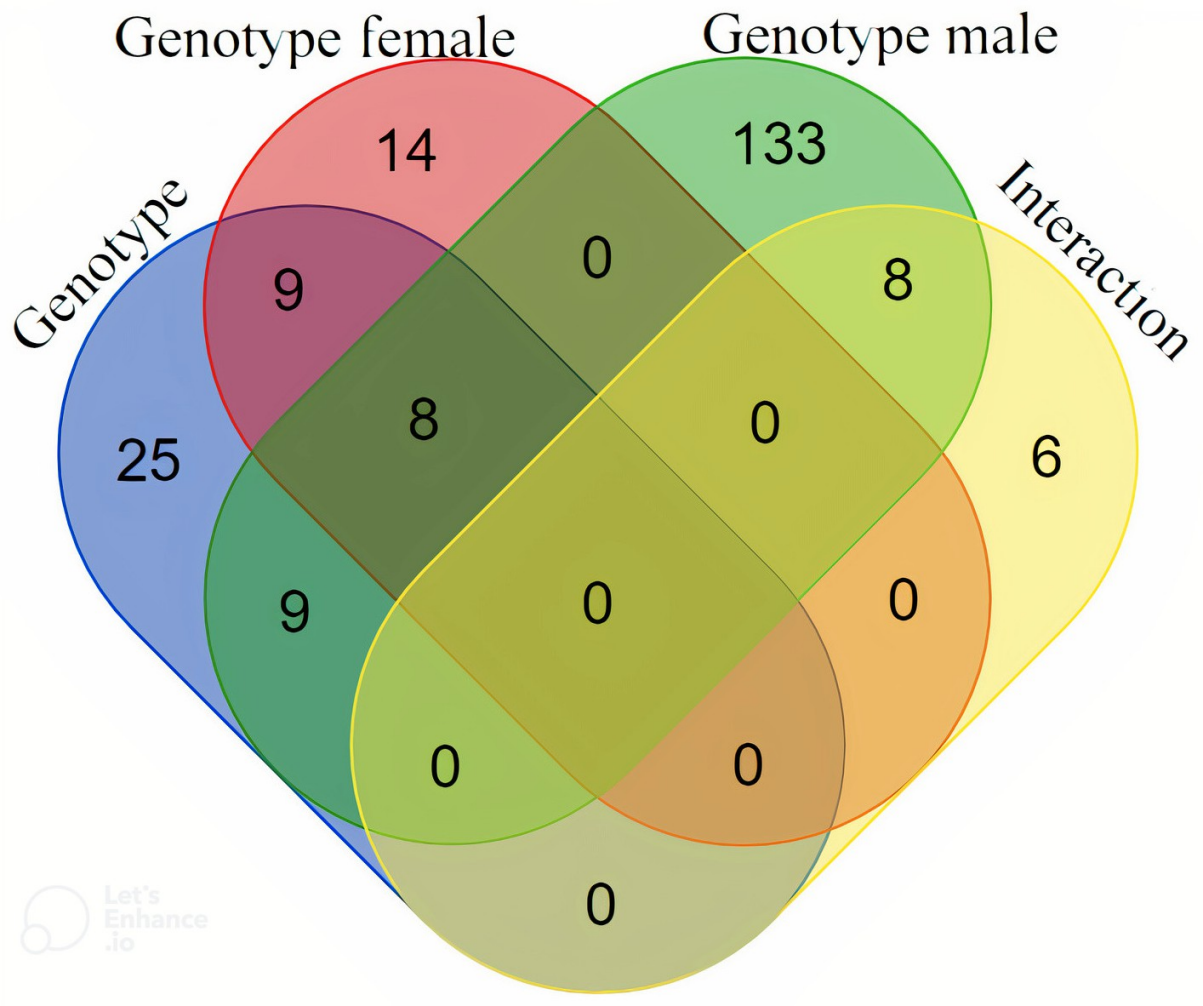
Venn diagram of the analyses of the genotype effect performed in the present study. Genotype: Hypothalamic transcriptome analysis of all IBxIB vs. all IBxLW 60 days-old pigs; Genotype females: Hypothalamic transcriptome analysis of IBxIB vs. IBxLW 60 days-old females; Genotype males: Hypothalamic transcriptome analysis of IBxIB vs. IBxLW 60 days-old males; Interaction: Hypothalamic transcriptome analysis of the genotype by sex interaction in 60 days-old pigs. IBxIB = pure Iberian pigs; IBxLW = Iberian x Large White crossbreds.

A comparative analysis was performed between results obtained in the studies of the genotype effect, the genotype effect within each sex and the interaction effect to fully understand them, which is summarized in Fig 4. Most genes were unique in each dataset, which further states the idea of the relevant differences found in each sex in the present study. However, the comparison of all IBxIB *vs* all IBxLW pigs shared 8 genes with both the analyses using only females and only males (*TBCD*, *TMEM138*, *CYR61*, *SLC6A9*, *DUT*, *TMEM9B*, *TACC3* and *SGCA)*. These 8 genes are those for which the genotype effect is clearest and independent of sex. The overall genotype analysis with all animals also shared 9 genes with the comparison IBxIB *vs*. IBxLW females (*PRR19*, *PGGHG*, *CHD8*, *USP16*, *GYPCÂ*, *SEPTIN1*, *TARBP1*, *CNOT7* and *FAM160B2)* and other 9 with the analysis comparing only males *(WDR45B*, *TMEM120B*, *LRRC45*, *CCDC84*, *CPSF7*, *PDZK1*, *ADCYAP1*, *KRR1* and *SMAP1)*. Moreover, the datasets from the comparison of IBxIB vs. IBxLW males and the interaction also had 8 genes in common (*LIN37*, *RBM12B*, *PLPP1*, *CELSR1*, *PSAP*, *DYNC1H1*, *GRPEL2* and *TMEM165)*, for which the evidence of a male-specific effect is stronger. Some of them had, as stated before, important implications in explaining phenotypic differences between IBxIB and IBxLW animals, like SGCA, GRPEL2 or TBCD, which may explain the coincidences among analyses.

### Result validation with qPCR

To validate the RNA-seq results, the relative expression of a selected group of DEGs was assessed (S7 Table). Due to the particularities of the results obtained, with substantial effect of sex in the differences of the transcriptome between genotypes, the genes selected for validation were differentially expressed in one of the following analyses: all animals, females, or males. Therefore, the RNA-seq and real time qPCR correlation was calculated separately for each subsampling group. As expected, in the group where the gene showed the higher difference of expression, the correlation showed high values, whereas poorer results were obtained when the difference of expression in the RNA-seq analyses was lower or non-significant.

As found in other studies [38, 124], differences tend to be more notable with the RNA-seq approach than with the Real-Time qPCR. However, the concordance correlation coefficient (CCC) showed an acceptable correlation between techniques (CCC in all samples = 0.73; CCC in females = 0.66; CCC in males = 0.60).

## Conclusions

Differences between 60 days-old pure Iberian and Iberian x Large White crossbreds were found at the hypothalamic transcriptome level, even when bred under the same prenatal and early postnatal environments. In accordance with the phenotype data, genotype effect on transcriptome was greater in males than in females. Given the importance of the hypothalamus in growth patterns and adiposity, these findings help to better understand the metabolic and physiological basis of phenotypic differences between lean and fatty pigs, which are important from a productive and biomedical point of view.

## Supporting information

S1 Fig PCA of the DEseq2 results separated by genotype (A) or by sex (B). PCAs comparing genotypes (A; red: IBxIB, blue: IBxLW) and sexes (B; red: females; blue: males).
(XLSX)

S1 Table Calculated analysis (g/kg, dry-matter basis) and fatty acid composition of the sows’ piglet’s diets. Nutrients and fatty acid composition of the diets used in the study.
(XLSX)

S2 Table qPCR primer design. qPCR primer designs.
(XLSX)

S3 Table Phenotypic differences (mean ± S.E.M.) between pure Iberian piglets (IBxIB) and Iberian x Large White crossbreds (IBxLW) at 60 days old of the animals selected for the present study. Table containing information regarding phenotypic differences of the animals selected in the present study.
(XLSX)

S4 Table Detailed list of DEGs using all animals when comparing pure Iberian pigs (IBxIB) and Iberian *Large White crossbreds (IBxLW). Table containing DEGs information using the total number of animals.
(XLSX)

S5 Table Detailed list of DEGs using females when comparing pure Iberian pigs (IBxIB) and Iberian *Large White crossbreds (IBxLW). Table containing DEGs information using a subset of the animals (only females).
(XLSX)

S6 Table Detailed list of DEGs using males when comparing pure Iberian pigs (IBxIB) and Iberian x Large White crossbreds (IBxLW). Table containing DEGs information using a subset of the animals (only males).
(XLSX)

S7 Table Validation of results obtained by DEseq2 using qPCR analyses. Bold data indicates the RNA-seq analysis in which the gene selected was significantly differentially expressed. Table containing validation results in the total number of animals, females and males analysis.
(XLSX)

## References

[1] Chang KC, da Costa N, Blackley R, Southwood O, Evans G, Plastow G, et al. Relationships of myosin heavy chain fibre types to meat quality traits in traditional and modern pigs. Meat Sci. 2003;64(1):93–103. Epub 2003/05/01. doi: 10.1016/s0309-1740(02)00208-5 . 22062667

[2] Kerr JC, Cameron ND. Reproductive performance of pigs selected for components of efficient lean growth. Animal Science. 1995;60(2):281–90. Epub 2010/09/02. doi: 10.1017/S1357729800008444

[3] Gonzalez-Añover P, Encinas T, Gomez-Izquierdo E, Sanz E, Letelier C, Torres-Rovira L, et al. Advanced Onset of Puberty in Gilts of Thrifty Genotype (Iberian Pig). Reproduction in Domestic Animals. 2010;45(6):1003–7. doi: 10.1111/j.1439-0531.2009.01476.x 19473306

[4] Lopez-Bote CJ. Sustained utilization of the Iberian pig breed. Meat Science. 1998;49:S17–S27. doi: 10.1016/S0309-1740(98)90036-5 22060709

[5] Pugliese C, Sirtori F. Quality of meat and meat products produced from southern European pig breeds. Meat Science. 2012;90(3):511–8. doi: 10.1016/j.meatsci.2011.09.019 22030111

[6] Ventanas S VJRCJEM. Iberian pigs for the development of high-quality cured products: Research Signpost; 2005 2020-10-26.

[7] Aigner B, Kessler B, Klymiuk N, Kurome M, Renner S, Wünsch A, et al. Chapter 26—Genetically Tailored Pig Models for Translational Biomedical Research. In: Conn PM, editor. Animal Models for the Study of Human Disease (Second Edition): Academic Press; 2017. p. 671–701.

[8] Torres-Rovira L, Astiz S, Caro A, Lopez-Bote C, Ovilo C, Pallares P, et al. Diet-Induced Swine Model with Obesity/Leptin Resistance for the Study of Metabolic Syndrome and Type 2 Diabetes. The Scientific World Journal. 2012;2012. doi: 10.1100/2012/510149 22629144 PMC3354447

[9] Torres-Rovira L, Gonzalez-Anover P, Astiz S, Caro A, Lopez-Bote C, Ovilo C, et al. Effect of an Obesogenic Diet During the Juvenile Period on Growth Pattern, Fatness and Metabolic, Cardiovascular and Reproductive Features of Swine with Obesity/Leptin Resistance. Endocrine‚ Metabolic & Immune Disorders-Drug Targets. 2013;13(2):143–51. doi: 10.2174/1871530311313020002 23094796

[10] Pérez-Montarelo D, Madsen O, Alves E, Rodríguez MC, Folch JM, Noguera JL, et al. Identification of genes regulating growth and fatness traits in pig through hypothalamic transcriptome analysis. Physiological genomics. 2014;46(6):195–206. Epub 2013/11/26. doi: 10.1152/physiolgenomics.00151.2013 . 24280257 PMC3949103

[11] Cone RD, Cowley MA, Butler AA, Fan W, Marks DL, Low MJ. The arcuate nucleus as a conduit for diverse signals relevant to energy homeostasis. International Journal of Obesity. 2001;25(5):S63–S7. doi: 10.1038/sj.ijo.0801913 11840218

[12] Lenard NR, Berthoud HR. Central and peripheral regulation of food intake and physical activity: pathways and genes. Obesity (Silver Spring). 2008;16 Suppl 3(Suppl 3):S11–22. Epub 2009/02/20. doi: 10.1038/oby.2008.511 . 19190620 PMC2687326

[13] Hou Y, Hu M, Zhou H, Li C, Li X, Liu X, et al. Neuronal Signal Transduction-Involved Genes in Pig Hypothalamus Affect Feed Efficiency as Revealed by Transcriptome Analysis. Biomed Res Int. 2018;2018:5862571-. doi: 10.1155/2018/5862571 . 30687750 PMC6327278

[14] Gley K, Murani E, Trakooljul N, Zebunke M, Puppe B, Wimmers K, et al. Transcriptome profiles of hypothalamus and adrenal gland linked to haplotype related to coping behavior in pigs. Sci Rep. 2019;9(1):13038. Epub 2019/09/12. doi: 10.1038/s41598-019-49521-2 . 31506580 PMC6736951

[15] Yuan X, Zhou X, Chen Z, He Y, Kong Y, Ye S, et al. Genome-Wide DNA Methylation Analysis of Hypothalamus During the Onset of Puberty in Gilts. Front Genet. 2019;10:228. Epub 2019/04/04. doi: 10.3389/fgene.2019.00228 . 30941164 PMC6433709

[16] Tan S, Zhou Y, Zhao H, Wu J, Yu H, Yang Y, et al. Comprehensive transcriptome analysis of hypothalamus reveals genes associated with disorders of sex development in pigs. J Steroid Biochem Mol Biol. 2021;210:105875. Epub 2021/03/23. doi: 10.1016/j.jsbmb.2021.105875 . 33746111

[17] Óvilo C, Fernández AIA, Fernández AIA, Folch JM, Varona L, Benítez R, et al. Hypothalamic expression of porcine leptin receptor (LEPR), neuropeptide y (NPY), and cocaine- and amphetamine-regulated transcript (CART) genes is influenced by LEPR genotype. Mammalian Genome. 2010;21(11–12):583–91. doi: 10.1007/s00335-010-9307-1 21128076

[18] Liu J, Cao S, Liu M, Chen L, Zhang H. A high nutrient dense diet alters hypothalamic gene expressions to influence energy intake in pigs born with low birth weight. Scientific Reports. 2018;8(1):5514. doi: 10.1038/s41598-018-23926-x 29615796 PMC5882958

[19] Piórkowska K, Żukowski K, Tyra M, Szyndler-Nędza M, Szulc K, Skrzypczak E, et al. The Pituitary Transcriptional Response Related to Feed Conversion in Pigs. Genes. 2019;10(9):712. doi: 10.3390/genes10090712 31540087 PMC6771146

[20] Cirera S, Jensen MS, Elbrønd VS, Moesgaard SG, Christoffersen BØ, Kadarmideen HN, et al. Expression studies of six human obesity-related genes in seven tissues from divergent pig breeds. Animal Genetics. 2014;45(1):59–66. doi: 10.1111/age.12082 24033492

[21] Wang Z, Gerstein M, Snyder M. RNA-Seq: a revolutionary tool for transcriptomics. Nature Reviews Genetics. 2009;10(1):57–63. doi: 10.1038/nrg2484 19015660 PMC2949280

[22] Cogollos L, Garcia-Contreras C, Vazquez-Gomez M, Astiz S, Sanchez-Sanchez R, Gomez-Fidalgo E, et al. Effects of fetal genotype and sex on developmental response to maternal malnutrition. Reproduction, Fertility and Development. 2017;29(6):1155–68. doi: 10.1071/RD15385 27184893

[23] García-Contreras C, Madsen O, Groenen MAM, López-García A, Vázquez-Gómez M, Astiz S, et al. Impact of genotype, body weight and sex on the prenatal muscle transcriptome of Iberian pigs. PLoS One. 2020;15(1):e0227861. Epub 2020/01/29. doi: 10.1371/journal.pone.0227861 however, this does not alter the authors’ adherence to all PLOS One policies. 31990923 PMC6986718

[24] Heras-Molina A, Pesantez JL, Astiz S, Garcia-Contreras C, Vazquez-Gomez M, Isabel B, et al. The Role of Offspring Genotype-By-Sex Interactions, Independently of Environmental Cues, on the Phenotype Traits of an Obese Swine Model. Biology. 2020;9(12):445. doi: 10.3390/biology9120445 . 33291637 PMC7761963

[25] Óvilo C, Fernández A, Noguera JL, Barragán C, Letón R, Rodríguez C, et al. Fine mapping of porcine chromosome 6 QTL and LEPR effects on body composition in multiple generations of an Iberian by Landrace intercross. Genetical Research. 2005;85(1):57–67. doi: 10.1017/s0016672305007330 16089036

[26] Council NR. Nutrient Requirements of Swine: Eleventh Revised Edition. Washington, DC: The National Academies Press; 2012. 420 p.

[27] Babraham Bioinformatics—FastQC A Quality Control tool for High Throughput Sequence Data 2021 [18 of May, 2021]. https://www.bioinformatics.babraham.ac.uk/projects/fastqc/.

[28] Babraham Bioinformatics—Trim Galore! 2021 [18 of May, 2021]. https://www.bioinformatics.babraham.ac.uk/projects/trim_galore/.

[29] Kim D, Paggi JM, Park C, Bennett C, Salzberg SL. Graph-based genome alignment and genotyping with HISAT2 and HISAT-genotype. Nature Biotechnology. 2019;37(8):907–15. doi: 10.1038/s41587-019-0201-4 31375807 PMC7605509

[30] Li H, Handsaker B, Wysoker A, Fennell T, Ruan J, Homer N, et al. The Sequence Alignment/Map format and SAMtools. Bioinformatics. 2009;25(16):2078–9. Epub 2009/06/10. doi: 10.1093/bioinformatics/btp352 . 19505943 PMC2723002

[31] Anders S, Pyl PT, Huber W. HTSeq—a Python framework to work with high-throughput sequencing data. Bioinformatics. 2014;31(2):166–9. doi: 10.1093/bioinformatics/btu638 25260700 PMC4287950

[32] Love MI, Huber W, Anders S. Moderated estimation of fold change and dispersion for RNA-seq data with DESeq2. Genome Biology. 2014;15(12):550. doi: 10.1186/s13059-014-0550-8 25516281 PMC4302049

[33] Home—QIAGEN Digital Insights 2021 [18 of May, 2021]. https://digitalinsights.qiagen.com/.

[34] STRING: functional protein association networks 2021 [18 of May, 2021]. https://string-db.org/.

[35] Szklarczyk D, Gable AL, Lyon D, Junge A, Wyder S, Huerta-Cepas J, et al. STRING v11: protein-protein association networks with increased coverage, supporting functional discovery in genome-wide experimental datasets. Nucleic Acids Res. 2019;47(D1):D607–d13. Epub 2018/11/27. doi: 10.1093/nar/gky1131 . 30476243 PMC6323986

[36] Krämer A, Green J, Pollard J Jr., Tugendreich S. Causal analysis approaches in Ingenuity Pathway Analysis. Bioinformatics. 2014;30(4):523–30. Epub 2013/12/18. doi: 10.1093/bioinformatics/btt703 . 24336805 PMC3928520

[37] Núñez Y, Radović Č, Savić R, García-Casco JM, Čandek-Potokar M, Benítez R, et al. Muscle Transcriptome Analysis Reveals Molecular Pathways Related to Oxidative Phosphorylation, Antioxidant Defense, Fatness and Growth in Mangalitsa and Moravka Pigs. Animals (Basel). 2021;11(3). Epub 2021/04/04. doi: 10.3390/ani11030844 . 33809803 PMC8002519

[38] Albuquerque A, Óvilo C, Núñez Y, Benítez R, López-Garcia A, García F, et al. Transcriptomic Profiling of Skeletal Muscle Reveals Candidate Genes Influencing Muscle Growth and Associated Lipid Composition in Portuguese Local Pig Breeds. Animals. 2021;11(5):1423. doi: 10.3390/ani11051423 34065673 PMC8156922

[39] Albuquerque A, Óvilo C, Núñez Y, Benítez R, López-Garcia A, García F, et al. Comparative Transcriptomic Analysis of Subcutaneous Adipose Tissue from Local Pig Breeds. Genes (Basel). 2020;11(4). Epub 2020/04/25. doi: 10.3390/genes11040422 . 32326415 PMC7231169

[40] Trexler M, Bányai L, Patthy L. A human protein containing multiple types of protease-inhibitory modules. Proc Natl Acad Sci U S A. 2001;98(7):3705–9. Epub 2001/03/29. doi: 10.1073/pnas.061028398 . 11274388 PMC31116

[41] Kondás K, Szláma G, Trexler M, Patthy L. Both WFIKKN1 and WFIKKN2 have high affinity for growth and differentiation factors 8 and 11. J Biol Chem. 2008;283(35):23677–84. Epub 2008/07/01. doi: 10.1074/jbc.M803025200 . 18596030 PMC3259755

[42] Kondás K, Szláma G, Nagy A, Trexler M, Patthy L. Biological functions of the WAP domain-containing multidomain proteins WFIKKN1 and WFIKKN2. Biochemical Society Transactions. 2011;39(5):1416–20. doi: 10.1042/BST0391416 21936825

[43] Szláma G, Kondás K, Trexler M, Patthy L. WFIKKN1 and WFIKKN2 bind growth factors TGFβ1, BMP2 and BMP4 but do not inhibit their signalling activity. Febs j. 2010;277(24):5040–50. Epub 2010/11/09. doi: 10.1111/j.1742-4658.2010.07909.x . 21054789

[44] Database GHG. KY Gene—GeneCards | KY Protein | KY Antibody. 2021.

[45] Contreras CG, Vazquez-Gomez M, Madsen O, Groenen M, Astiz S, Nunez Y, et al., editors. Fetal genotype effects on morphomics, fatty acids composition and transcriptomics in swine. ISAG 2019 Abstract Book; 2019.

[46] Benítez R, Trakooljul N, Núñez Y, Isabel B, Murani E, De Mercado E, et al. Breed, Diet, and Interaction Effects on Adipose Tissue Transcriptome in Iberian and Duroc Pigs Fed Different Energy Sources. Genes. 2019;10(8):589. doi: 10.3390/genes10080589 31382709 PMC6723240

[47] Temme C, Zaessinger S, Meyer S, Simonelig M, Wahle E. A complex containing the CCR4 and CAF1 proteins is involved in mRNA deadenylation in Drosophila. EMBO J. 2004;23(14):2862–71. doi: 10.1038/sj.emboj.7600273 . 15215893 PMC514940

[48] Bianchin C, Mauxion F, Sentis S, Séraphin B, Corbo L. Conservation of the deadenylase activity of proteins of the Caf1 family in human. RNA. 2005;11(4):487–94. doi: 10.1261/rna.7135305 . 15769875 PMC1370737

[49] Weill L, Belloc E, Bava FA, Méndez R. Translational control by changes in poly(A) tail length: recycling mRNAs. Nat Struct Mol Biol. 2012;19(6):577–85. Epub 2012/06/06. doi: 10.1038/nsmb.2311 . 22664985

[50] Yu N, Zhang P, Wang L, He X, Yang S, Lu H. RBBP7 is a prognostic biomarker in patients with esophageal squamous cell carcinoma. Oncol Lett. 2018;16(6):7204–11. Epub 2018/10/03. doi: 10.3892/ol.2018.9543 . 30546458 PMC6256704

[51] Giri R, Yeh H-H, Wu C-H, Liu H-S. SUMO-1 overexpression increases RbAp46 protein stability and suppresses cell growth. Anticancer research. 2008;28(6A):3749–56. 19189660

[52] Piras IS, Krate J, Delvaux E, Nolz J, Mastroeni DF, Persico AM, et al. Transcriptome changes in the Alzheimer’s disease middle temporal gyrus: importance of RNA metabolism and mitochondria-associated membrane genes. Journal of Alzheimer’s Disease. 2019;70(3):691–713. doi: 10.3233/JAD-181113 31256118

[53] Beach TG, Adler CH, Sue LI, Serrano G, Shill HA, Walker DG, et al. Arizona Study of Aging and Neurodegenerative Disorders and Brain and Body Donation Program. Neuropathology. 2015;35(4):354–89. Epub 2015/01/27. doi: 10.1111/neup.12189 . 25619230 PMC4593391

[54] Baxter EM, Jarvis S, Palarea-Albaladejo J, Edwards SA. The weaker sex? The propensity for male-biased piglet mortality. PLoS One. 2012;7(1):e30318. Epub 2012/01/25. doi: 10.1371/journal.pone.0030318 . 22272334 PMC3260262

[55] Bæk O, Cilieborg MS, Nguyen DN, Bering SB, Thymann T, Sangild PT. Sex-Specific Survival, Growth, Immunity and Organ Development in Preterm Pigs as Models for Immature Newborns. Frontiers in Pediatrics. 2021;9(63). doi: 10.3389/fped.2021.626101 33643975 PMC7905020

[56] Gonzalez-Bulnes A, Ovilo C, Lopez-Bote CJ, Astiz S, Ayuso M, Perez-Solana ML, et al. Gender-specific early postnatal catch-up growth after intrauterine growth retardation by food restriction in swine with obesity/leptin resistance. Reproduction. 2012;144(2):269–78. doi: 10.1530/REP-12-0105 22692087

[57] Jacob RM, Mudd AT, Alexander LS, Lai C-S, Dilger RN. Comparison of Brain Development in Sow-Reared and Artificially Reared Piglets. Frontiers in Pediatrics. 2016;4(95). doi: 10.3389/fped.2016.00095 27672632 PMC5018487

[58] Konovalova S, Liu X, Manjunath P, Baral S, Neupane N, Hilander T, et al. Redox regulation of GRPEL2 nucleotide exchange factor for mitochondrial HSP70 chaperone. Redox Biology. 2018;19:37–45. doi: 10.1016/j.redox.2018.07.024 30098457 PMC6089081

[59] Srivastava S, Savanur MA, Sinha D, Birje A, R V, Saha PP, et al. Regulation of mitochondrial protein import by the nucleotide exchange factors GrpEL1 and GrpEL2 in human cells. J Biol Chem. 2017;292(44):18075–90. Epub 2017/08/30. doi: 10.1074/jbc.M117.788463 . 28848044 PMC5672033

[60] Tsikas D. Assessment of lipid peroxidation by measuring malondialdehyde (MDA) and relatives in biological samples: Analytical and biological challenges. Analytical Biochemistry. 2017;524:13–30. doi: 10.1016/j.ab.2016.10.021 27789233

[61] Wang W, Zhao LJ, Yang Y, Wang RY, Ren H, Zhao P, et al. Retinoic acid induced 16 enhances tumorigenesis and serves as a novel tumor marker for hepatocellular carcinoma. Carcinogenesis. 2012;33(12):2578–85. Epub 2012/09/14. doi: 10.1093/carcin/bgs289 . 22971576

[62] Qian CL, Ding CL, Tang HL, Qi ZT, Wang W. Retinoic acid induced 16 deficiency exacerbates high-fat diet-induced steatohepatitis in mice. Cell Biochem Funct. 2020;38(6):753–60. Epub 2020/04/15. doi: 10.1002/cbf.3542 . 32289885

[63] Ding CL, Qian CL, Qi ZT, Wang W. Identification of retinoid acid induced 16 as a novel androgen receptor target in prostate cancer cells. Mol Cell Endocrinol. 2020;506:110745. Epub 2020/02/06. doi: 10.1016/j.mce.2020.110745 . 32014455

[64] Mavioğlu RN, Kara B, Akansel G, Nalbant G, Tolun A. FAM160B1 deficit associated with microcephaly, severe intellectual disability, ataxia, behavioral abnormalities and speech problems. Clin Genet. 2019;96(5):456–60. Epub 2019/07/30. doi: 10.1111/cge.13612 . 31353455

[65] Zhang Y, Liu RB, Cao Q, Fan KQ, Huang LJ, Yu JS, et al. USP16-mediated deubiquitination of calcineurin A controls peripheral T cell maintenance. J Clin Invest. 2019;129(7):2856–71. Epub 2019/05/29. doi: 10.1172/JCI123801 . 31135381 PMC6597231

[66] Li Y, Rao Y, Zhu H, Jiang B, Zhu M. USP16 Regulates the Stability and Function of LDL receptor by Deubiquitination. Int Heart J. 2020;61(5):1034–40. Epub 2020/10/02. doi: 10.1536/ihj.20-043 . 32999190

[67] Rogge MM. The role of impaired mitochondrial lipid oxidation in obesity. Biol Res Nurs. 2009;10(4):356–73. Epub 2009/02/05. doi: 10.1177/1099800408329408 . 19190032

[68] Martínez-Reyes I, Chandel NS. Mitochondrial TCA cycle metabolites control physiology and disease. Nature Communications. 2020;11(1):102. doi: 10.1038/s41467-019-13668-3 31900386 PMC6941980

[69] Xu Y, Li L, Li J, Liu Q. Structural and biological function of NYD-SP15 as a new member of cytidine deaminases. Gene. 2016;583(1):36–47. Epub 2016/03/08. doi: 10.1016/j.gene.2016.02.048 . 26945630

[70] Liu Q, Liu J, Cao Q, Sha J, Zhou Z, Wang H, et al. NYD-SP15: a novel gene potentially involved in regulating testicular development and spermatogenesis. Biochem Genet. 2006;44(7–8):409–23. Epub 2006/09/07. doi: 10.1007/s10528-006-9038-x . 16955368

[71] Carvajal G, Brukman NG, Weigel Muñoz M, Battistone MA, Guazzone VA, Ikawa M, et al. Impaired male fertility and abnormal epididymal epithelium differentiation in mice lacking CRISP1 and CRISP4. Scientific Reports. 2018;8(1):17531. doi: 10.1038/s41598-018-35719-3 30510210 PMC6277452

[72] Tian G, Thomas S, Cowan NJ. Effect of TBCD and its regulatory interactor Arl2 on tubulin and microtubule integrity. Cytoskeleton (Hoboken). 2010;67(11):706–14. doi: 10.1002/cm.20480 . 20740604 PMC2958230

[73] Miyake N, Fukai R, Ohba C, Chihara T, Miura M, Shimizu H, et al. Biallelic TBCD Mutations Cause Early-Onset Neurodegenerative Encephalopathy. Am J Hum Genet. 2016;99(4):950–61. Epub 2016/09/27. doi: 10.1016/j.ajhg.2016.08.005 . 27666374 PMC5065661

[74] Tian D, Rizwan K, Liu Y, Kang L, Yang Y, Mao X, et al. Biallelic pathogenic variants in TBCD-related neurodevelopment disease with mild clinical features. Neurol Sci. 2019;40(11):2325–31. Epub 2019/06/27. doi: 10.1007/s10072-019-03979-0 . 31240573

[75] Amador-Licona N, Martinez-Cordero C, Guizar-Mendoza JM, Malacara JM, Hernandez J, Alcalá JF. Catch-up growth in infants born small for gestational age-a longitudinal study. The Journal of pediatric endocrinology. 2007;20(3):379. doi: 10.1515/jpem.2007.20.3.379 17451076

[76] Wright TM, Fone KCF, Langley-Evans SC, Voigt J-PW. Exposure to maternal consumption of cafeteria diet during the lactation period programmes feeding behaviour in the rat. International Journal of Developmental Neuroscience. 2011;29(8):785–93. doi: 10.1016/j.ijdevneu.2011.09.007 22004940

[77] Oyhenart E, Orden B, Fucini M, Muñe M, Pucciarelli H. Sexual dimorphism and postnatal growth of intrauterine growth retarded rats. Growth, Development, and Aging. 2003;67:73–83. 14535535

[78] Berg JM TJ, Stryer L. Biochemistry. Oxidative Phosphorylation. Biochemistry. New York: W H Freeman; 2002.

[79] Anderson S, Bankier AT, Barrell BG, de Bruijn MHL, Coulson AR, Drouin J, et al. Sequence and organization of the human mitochondrial genome. Nature. 1981;290(5806):457–65. doi: 10.1038/290457a0 7219534

[80] Sharma LK, Lu J, Bai Y. Mitochondrial respiratory complex I: structure, function and implication in human diseases. Curr Med Chem. 2009;16(10):1266–77. doi: 10.2174/092986709787846578 . 19355884 PMC4706149

[81] Schägger H, Pfeiffer K. Supercomplexes in the respiratory chains of yeast and mammalian mitochondria. EMBO J. 2000;19(8):1777–83. Epub 2000/04/25. doi: 10.1093/emboj/19.8.1777 . 10775262 PMC302020

[82] Iwata S, Lee JW, Okada K, Lee JK, Iwata M, Rasmussen B, et al. Complete structure of the 11-subunit bovine mitochondrial cytochrome bc1 complex. Science. 1998;281(5373):64–71. Epub 1998/07/04. doi: 10.1126/science.281.5373.64 . 9651245

[83] Michel H, Behr J, Harrenga A, Kannt A. Cytochrome c oxidase: structure and spectroscopy. Annu Rev Biophys Biomol Struct. 1998;27:329–56. Epub 1998/07/01. doi: 10.1146/annurev.biophys.27.1.329 . 9646871

[84] Yoshikawa S, Muramoto K, Shinzawa-Itoh K, Aoyama H, Tsukihara T, Shimokata K, et al. Proton pumping mechanism of bovine heart cytochrome c oxidase. Biochim Biophys Acta. 2006;1757(9–10):1110–6. Epub 2006/08/15. doi: 10.1016/j.bbabio.2006.06.004 . 16904626

[85] Jonckheere AI, Smeitink JA, Rodenburg RJ. Mitochondrial ATP synthase: architecture, function and pathology. J Inherit Metab Dis. 2012;35(2):211–25. Epub 2011/08/30. doi: 10.1007/s10545-011-9382-9 . 21874297 PMC3278611

[86] Misu H, Takamura T, Matsuzawa N, Shimizu A, Ota T, Sakurai M, et al. Genes involved in oxidative phosphorylation are coordinately upregulated with fasting hyperglycaemia in livers of patients with type 2 diabetes. Diabetologia. 2007;50(2):268–77. Epub 2006/12/26. doi: 10.1007/s00125-006-0489-8 . 17187250

[87] Dahlman I, Forsgren M, Sjögren A, Nordström EA, Kaaman M, Näslund E, et al. Downregulation of electron transport chain genes in visceral adipose tissue in type 2 diabetes independent of obesity and possibly involving tumor necrosis factor-alpha. Diabetes. 2006;55(6):1792–9. Epub 2006/05/30. doi: 10.2337/db05-1421 . 16731844

[88] Patti ME, Butte AJ, Crunkhorn S, Cusi K, Berria R, Kashyap S, et al. Coordinated reduction of genes of oxidative metabolism in humans with insulin resistance and diabetes: Potential role of PGC1 and NRF1. Proc Natl Acad Sci U S A. 2003;100(14):8466–71. Epub 2003/07/02. doi: 10.1073/pnas.1032913100 . 12832613 PMC166252

[89] Nelson DL, Lehninger AL, Cox MM, Foix CMC, León S, Roca JV. Lehninger principios de bioquímica: Omega; 2013.

[90] Balaban RS, Nemoto S, Finkel T. Mitochondria, oxidants, and aging. cell. 2005;120(4):483–95. doi: 10.1016/j.cell.2005.02.001 15734681

[91] Alfadda AA, Sallam RM. Reactive oxygen species in health and disease. J Biomed Biotechnol. 2012;2012:936486. Epub 2012/08/29. doi: 10.1155/2012/936486 . 22927725 PMC3424049

[92] Chatterjee S. Chapter Two—Oxidative Stress, Inflammation, and Disease. In: Dziubla T, Butterfield DA, editors. Oxidative Stress and Biomaterials: Academic Press; 2016. p. 35–58.

[93] Aroor AR, DeMarco VG. Oxidative Stress and Obesity: The Chicken or the Egg? Diabetes. 2014;63(7):2216–8. doi: 10.2337/db14-0424 24962921

[94] Lee CH, Kim HJ, Lee Y-S, Kang GM, Lim HS, Lee S-H, et al. Hypothalamic Macrophage Inducible Nitric Oxide Synthase Mediates Obesity-Associated Hypothalamic Inflammation. Cell Rep. 2018;25(4):934–46.e5. doi: 10.1016/j.celrep.2018.09.070 . 30355499 PMC6284237

[95] Slauch JM. How does the oxidative burst of macrophages kill bacteria? Still an open question. Molecular Microbiology. 2011;80(3):580–3. doi: 10.1111/j.1365-2958.2011.07612.x 21375590 PMC3109634

[96] Kohchi C, Inagawa H, Nishizawa T, Soma G-I. ROS and innate immunity. Anticancer research. 2009;29(3):817–21. 19414314

[97] Takamura T, Misu H, Matsuzawa-Nagata N, Sakurai M, Ota T, Shimizu A, et al. Obesity upregulates genes involved in oxidative phosphorylation in livers of diabetic patients. Obesity (Silver Spring). 2008;16(12):2601–9. Epub 2008/10/11. doi: 10.1038/oby.2008.419 . 18846047

[98] Buchner DA, Yazbek SN, Solinas P, Burrage LC, Morgan MG, Hoppel CL, et al. Increased mitochondrial oxidative phosphorylation in the liver is associated with obesity and insulin resistance. Obesity (Silver Spring). 2011;19(5):917–24. Epub 2010/10/05. doi: 10.1038/oby.2010.214 . 20885388 PMC3749733

[99] Dandoy-Dron F, Itier J-M, Monthioux E, Bucchini D, Jami J. Tissue-specific expression of the rat insulin 1 gene in vivo requires both the enhancer and promoter regions. Differentiation. 1995;58(4):291–5. doi: 10.1046/j.1432-0436.1995.5840291.x 7641979

[100] Sano S, Itami S, Takeda K, Tarutani M, Yamaguchi Y, Miura H, et al. Keratinocyte‐specific ablation of Stat3 exhibits impaired skin remodeling, but does not affect skin morphogenesis. The EMBO journal. 1999;18(17):4657–68. doi: 10.1093/emboj/18.17.4657 10469645 PMC1171539

[101] Cui Y, Huang L, Elefteriou F, Yang G, Shelton JM, Giles JE, et al. Essential role of STAT3 in body weight and glucose homeostasis. Molecular and cellular biology. 2004;24(1):258–69. doi: 10.1128/MCB.24.1.258-269.2004 14673160 PMC303343

[102] Akira S, Nishio Y, Inoue M, Wang X-J, We S, Matsusaka T, et al. Molecular cloning of APRF, a novel IFN-stimulated gene factor 3 p91-related transcription factor involved in the gp130-mediated signaling pathway. Cell. 1994;77(1):63–71. doi: 10.1016/0092-8674(94)90235-6 7512451

[103] Liu H, Du T, Li C, Yang G. STAT3 phosphorylation in central leptin resistance. Nutrition & Metabolism. 2021;18(1):39. doi: 10.1186/s12986-021-00569-w 33849593 PMC8045279

[104] Bates SH, Stearns WH, Dundon TA, Schubert M, Tso AW, Wang Y, et al. STAT3 signalling is required for leptin regulation of energy balance but not reproduction. Nature. 2003;421(6925):856–9. doi: 10.1038/nature01388 12594516

[105] Kitamura T, Feng Y, Kitamura YI, Chua SC Jr., Xu AW, Barsh GS, et al. Forkhead protein FoxO1 mediates Agrp-dependent effects of leptin on food intake. Nat Med. 2006;12(5):534–40. Epub 2006/04/11. doi: 10.1038/nm1392 . 16604086

[106] Cawley NX, Li Z, Loh YP. 60 YEARS OF POMC: Biosynthesis, trafficking, and secretion of pro-opiomelanocortin-derived peptides. Journal of molecular endocrinology. 2016;56(4):T77–T97. doi: 10.1530/JME-15-0323 26880796 PMC4899099

[107] Biebermann H, Castañeda TR, van Landeghem F, von Deimling A, Escher F, Brabant G, et al. A role for β-melanocyte-stimulating hormone in human body-weight regulation. Cell metabolism. 2006;3(2):141–6. doi: 10.1016/j.cmet.2006.01.007 16459315

[108] Bumaschny VF, Yamashita M, Casas-Cordero R, Otero-Corchón V, de Souza FS, Rubinstein M, et al. Obesity-programmed mice are rescued by early genetic intervention. The Journal of clinical investigation. 2012;122(11):4203–12. doi: 10.1172/JCI62543 23093774 PMC3484438

[109] Zhang Y, Fan G, Liu X, Skovgaard K, Sturek M, Heegaard PMH. The genome of the naturally evolved obesity-prone Ossabaw miniature pig. iScience. 2021;24(9):103081. doi: 10.1016/j.isci.2021.103081 34585119 PMC8455653

[110] Araujo EP, Moraes JC, Cintra DE, Velloso LA. MECHANISMS IN ENDOCRINOLOGY: Hypothalamic inflammation and nutrition. European Journal of Endocrinology. 2016;175(3):R97–R105. doi: 10.1530/EJE-15-1207 27006108

[111] Cakir I, Nillni EA. Endoplasmic reticulum stress, the hypothalamus, and energy balance. Trends in Endocrinology & Metabolism. 2019;30(3):163–76. 30691778 10.1016/j.tem.2019.01.002

[112] Schneeberger M, Dietrich MO, Sebastián D, Imbernón M, Castaño C, Garcia A, et al. Mitofusin 2 in POMC neurons connects ER stress with leptin resistance and energy imbalance. Cell. 2013;155(1):172–87. doi: 10.1016/j.cell.2013.09.003 24074867 PMC3839088

[113] Morales J, Pérez JF, Baucells MD, Mourot J, Gasa J. Comparative digestibility and lipogenic activity in Landrace and Iberian finishing pigs fed ad libitum corn- and corŽ sorghum acorn-based diets. Livestock Production Science. 2002;77:195–205.

[114] Carbognin E, Betto RM, Soriano ME, Smith AG, Martello G. Stat3 promotes mitochondrial transcription and oxidative respiration during maintenance and induction of naive pluripotency. The EMBO Journal. 2016;35(6):618–34. doi: 10.15252/embj.201592629 26903601 PMC4801951

[115] Haigis MC, Sinclair DA. Mammalian sirtuins: biological insights and disease relevance. Annual Review of Pathology: Mechanisms of Disease. 2010;5:253–95. doi: 10.1146/annurev.pathol.4.110807.092250 20078221 PMC2866163

[116] Lombard DB, Tishkoff DX, Bao J. Mitochondrial sirtuins in the regulation of mitochondrial activity and metabolic adaptation. Handb Exp Pharmacol. 2011;206:163–88. Epub 2011/09/01. doi: 10.1007/978-3-642-21631-2_8 . 21879450 PMC3245626

[117] Lombard DB, Alt FW, Cheng HL, Bunkenborg J, Streeper RS, Mostoslavsky R, et al. Mammalian Sir2 homolog SIRT3 regulates global mitochondrial lysine acetylation. Mol Cell Biol. 2007;27(24):8807–14. Epub 2007/10/10. doi: 10.1128/MCB.01636-07 . 17923681 PMC2169418

[118] Hirschey MD, Shimazu T, Goetzman E, Jing E, Schwer B, Lombard DB, et al. SIRT3 regulates mitochondrial fatty-acid oxidation by reversible enzyme deacetylation. Nature. 2010;464(7285):121–5. Epub 2010/03/06. doi: 10.1038/nature08778 . 20203611 PMC2841477

[119] Palacios OM, Carmona JJ, Michan S, Chen KY, Manabe Y, Ward JL 3rd, et al. Diet and exercise signals regulate SIRT3 and activate AMPK and PGC-1alpha in skeletal muscle. Aging (Albany NY). 2009;1(9):771–83. Epub 2010/02/17. doi: 10.18632/aging.100075 . 20157566 PMC2815736

[120] Schwer B, Eckersdorff M, Li Y, Silva JC, Fermin D, Kurtev MV, et al. Calorie restriction alters mitochondrial protein acetylation. Aging Cell. 2009;8(5):604–6. Epub 2009/07/15. doi: 10.1111/j.1474-9726.2009.00503.x . 19594485 PMC2752488

[121] Shi T, Wang F, Stieren E, Tong Q. SIRT3, a mitochondrial sirtuin deacetylase, regulates mitochondrial function and thermogenesis in brown adipocytes. J Biol Chem. 2005;280(14):13560–7. Epub 2005/01/18. doi: 10.1074/jbc.M414670200 . 15653680

[122] Kendrick AA, Choudhury M, Rahman SM, McCurdy CE, Friederich M, Van Hove JL, et al. Fatty liver is associated with reduced SIRT3 activity and mitochondrial protein hyperacetylation. Biochem J. 2011;433(3):505–14. Epub 2010/11/04. doi: 10.1042/BJ20100791 . 21044047 PMC3398511

[123] Bao J, Scott I, Lu Z, Pang L, Dimond CC, Gius D, et al. SIRT3 is regulated by nutrient excess and modulates hepatic susceptibility to lipotoxicity. Free Radical Biology and Medicine. 2010;49(7):1230–7. doi: 10.1016/j.freeradbiomed.2010.07.009 20647045 PMC2943385

[124] Ayuso M, Fernández A, Núñez Y, Benítez R, Isabel B, Barragán C, et al. Comparative analysis of muscle transcriptome between pig genotypes identifies genes and regulatory mechanisms associated to growth, fatness and metabolism. PloS one. 2015;10(12):e0145162. doi: 10.1371/journal.pone.0145162 26695515 PMC4687939

